# Lipidomic Signatures in Feline Disease: A PRISMA-Guided Systematic Review

**DOI:** 10.3390/metabo16050330

**Published:** 2026-05-15

**Authors:** Ana Carolina Fontes, Carolina Santos Silva, Ana Carolina Matos, Isabel Ribeiro Dias, Francisco Peixoto, Maria Manuel Oliveira, Maria Rosario Domingues, Carlos Antunes Viegas

**Affiliations:** 1 Department of Veterinary Sciences, School of Agricultural and Veterinary Sciences (ECAV), University of Trás-os-Montes and Alto Douro, 5000-801 Vila Real, Portugal; al61909@alunos.utad.pt (A.C.F.); al57891@alunos.utad.pt (C.S.S.); acarolinafmatos@gmail.com (A.C.M.); idias@utad.pt (I.R.D.); 2 Animal and Veterinary Research Center (CECAV)-AL4AnimalS, University of Trás-os-Montes and Alto Douro, 5000-801 Vila Real, Portugal; 3 Chemistry Center of Vila Real, University of Trás-os-Montes and Alto Douro, 5000-801 Vila Real, Portugal; fpeixoto@utad.pt (F.P.); mmso@utad.pt (M.M.O.); 4 RISE-Health—Health Research Network, Faculty of Medicine, University of Porto, 4099-002 Porto, Portugal; 5 CESAM, Departament of Chemistry, University of Aveiro, Campus Universitário de Santiago, 3810-193 Aveiro, Portugal; mrd@ua.pt; 6 Mass Spectrometry Centre, LAQV-REQUIMTE, Department of Chemistry, University of Aveiro, 3810-193 Aveiro, Portugal; 7 CIVG—Vasco da Gama Research Center, University School Vasco da Gama (EUVG), Campus Universitário, Avenida José Rodrigues Sousa Fernandes, 3020-210 Coimbra, Portugal

**Keywords:** fatty acids, feline disease, lipid profiling, lipid remodeling, lipid species, lipidomics, lipoproteins, metabolic biomarkers, oxylipins, phospholipids, sphingolipids, triglycerides

## Abstract

**Background/Objectives**: Lipidomics has become a key component of systems biology, enabling comprehensive characterisation of lipid species and their roles in health and disease. As regulators of membrane architecture, energy balance, inflammation, and cellular signalling, lipids offer a powerful framework for understanding metabolic dysfunction. In veterinary medicine, however, lipidomics remains comparatively underdeveloped. In cats, lipid metabolism is central to disorders such as hepatic lipidosis, cystitis, obesity, diabetes mellitus, and chronic inflammatory enteropathies, yet available data remain limited. This systematic review synthesised current evidence on lipidomics and lipid-focused profiling in feline disease and identified lipid alterations with potential clinical relevance. **Methods**: Following PRISMA 2020 guidelines, PubMed, ScienceDirect, and Scopus were searched for original studies (1994–2026) evaluating lipidomics or lipid-focused profiling in cats. Eligible studies assessed lipid species, fatty acids, lipid mediators, or lipoproteins in disease or physiological states. Owing to methodological heterogeneity, findings were synthesised narratively. **Results**: Seventeen studies met inclusion criteria, covering hepatic, urinary, gastrointestinal, renal, neurological, oncological, metabolic, and pharmacologically modulated conditions. Recurring alterations involved lipoproteins, triglycerides, phospholipids, sphingolipids, fatty acids, and oxylipins. More consistent patterns emerged in hepatic lipidosis, where lipoprotein disturbances may aid diagnosis; in lower urinary tract disease, where PUFA-derived oxylipins differentiated bacterial from idiopathic cystitis; and in obesity, where phospholipid and triglyceride shifts reflected metabolic risk. Fatty acid remodelling in chronic enteropathies aligned with mucosal inflammation, while sphingolipid changes in neurological disease correlated with severity. Heterogeneity in analytical platforms, dietary control, and study design limited comparability. **Conclusions**: Feline lipidomics reveals biologically meaningful alterations with emerging diagnostic and prognostic value. Although still developing, lipid-focused approaches may enhance disease characterisation and support translational research. Larger, standardised studies and robust reference datasets are needed to validate lipid signatures for clinical implementation.

## 1. Introduction

Lipids comprise a structurally diverse group of molecules that collectively form the lipidome of an organism, encompassing hundreds of thousands of distinct molecular species. Broadly, lipids are defined as organic compounds that are insoluble in water but soluble in organic solvents, a property shared by molecules with markedly different structural features. This extensive structural and functional diversity is reflected in the complexity of lipid classification systems [1].

The LIPID MAPS consortium classifies lipids into eight major categories: fatty acyls, glycerolipids, glycerophospholipids, sphingolipids, sterol lipids, prenol lipids, polyketides, and saccharolipids [1,2,3,4]. Each category is further subdivided into classes and subclasses, reflecting the wide diversity of lipid structures and biological functions.

The structural diversity of lipid molecules underpins their broad range of biological roles [1]. Lipids are essential components of cellular membranes, regulators of energy metabolism, and key mediators of both intracellular and extracellular signalling processes [5,6]. They contribute to membrane architecture, modulate the activity of membrane-associated proteins, and act as bioactive molecules involved in inflammatory and metabolic pathways.

Lipidomics has emerged as a central component of systems biology, enabling the comprehensive characterisation of lipid species and their roles in health and disease [7,8]. Because lipids regulate membrane structure, energy homeostasis, inflammation, and cellular signalling, lipidomic profiling has provided critical insights into a wide range of pathological conditions. In human medicine, lipidomics has been successfully applied to the study of metabolic disorders such as obesity and type 2 diabetes, where alterations in lipid species reflect early metabolic dysregulation; cardiovascular diseases, through the identification of lipid signatures associated with atherosclerosis; neurodegenerative disorders, including Alzheimer’s disease, where lipid imbalance contributes to neuronal dysfunction; and cancer, where lipid metabolism reprogramming supports tumour growth and progression [8,9,10,11,12,13,14,15,16,17].

**Figure 1:**
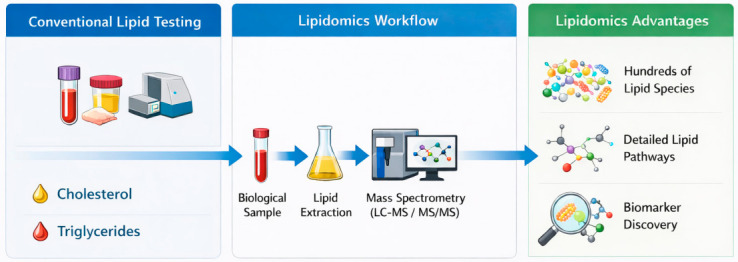
Overview of the lipidomics workflow and its advantages compared to conventional lipid analysis. Traditional approaches typically quantify a limited number of lipid classes (e.g., cholesterol and triglycerides), whereas lipidomics enables comprehensive profiling of hundreds of lipid species, providing detailed insights into metabolic pathways and biomarker discovery.

Advances in high-resolution mass spectrometry and bioinformatics have further accelerated the discovery of lipid biomarkers with diagnostic and prognostic value, positioning lipidomics as a key pillar of precision medicine and translational research [18]. A major strength of lipidomics lies in its ability to provide high-resolution, system-wide characterisation of lipid species, enabling the detection of subtle metabolic alterations that are not captured by conventional biochemical assays focused on total lipid concentrations. By integrating structural, functional, and pathway-level information, lipidomics offers a more comprehensive understanding of disease mechanisms and supports the identification of biologically meaningful biomarkers [5,6,19] as illustrated in Figure 1. Moreover, lipidomic approaches allow the simultaneous interrogation of multiple metabolic pathways, facilitating the identification of interconnected alterations that would otherwise remain undetected using traditional targeted analyses.

In veterinary medicine, lipidomics is increasingly recognised as a powerful tool for elucidating species-specific metabolic pathways and improving the diagnosis of naturally occurring diseases in companion animals, livestock, and wildlife. Comparative lipidomic studies have contributed to the characterisation of inflammatory, endocrine, metabolic, and nutritional disorders across species, reinforcing One Health perspectives and supporting translational research frameworks [20,21,22]. These approaches are particularly valuable in veterinary contexts, where interspecies differences limit the direct extrapolation of human data. Despite this potential, lipidomics remains underutilised in veterinary diagnostics, largely due to interspecies variability and the limited availability of robust reference datasets.

Among companion animals, the domestic cat remains one of the least explored species from a lipidomic perspective. Felines exhibit unique metabolic adaptations—including strict carnivory, limited carbohydrate metabolism, and distinctive fatty acid requirements—that profoundly shape their lipid profiles [23]. These species-specific characteristics make lipidomic approaches particularly relevant for advancing feline pathophysiology, as conventional extrapolation from other species may be inadequate. However, only a limited number of studies have characterised feline lipid classes or established baseline reference intervals [24,25]. This lack of foundational data constrains the interpretation of lipid alterations in disease and underscores the need for systematic, large-scale lipidomic mapping in cats.

In the present review, the term “lipidomics” is used in a broad but explicitly defined sense. Comprehensive mass spectrometry-based analyses were considered true lipidomics, whereas targeted lipid profiling approaches were included when they contributed directly to the understanding of disease-associated lipid alterations in cats. This distinction is important, as the feline literature spans a continuum from conventional lipid profiling to higher-resolution lipidomic methodologies. Accordingly, the field is interpreted here as lipidomics-informed rather than uniformly lipidomics-driven [5].

**Figure 2:**
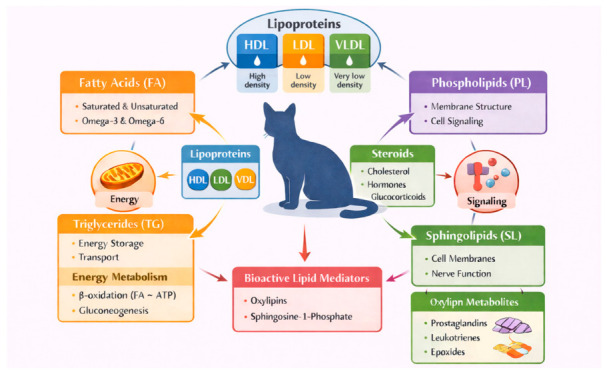
Overview of the feline lipidome: lipid classes and functional roles. This schematic summarises key lipid classes and their involvement in fundamental biological processes, including energy metabolism, membrane dynamics, and signalling pathways. The central depiction highlights the integrated and system-wide nature of lipid metabolism in cats.

Given the breadth of lipid classes and their involvement across multiple biological systems, lipid alterations in cats are expected to span diverse disease categories and physiological states (Figure 2).

This systematic review was conducted in accordance with the PRISMA 2020 guidelines [26] and aims to synthesise the available evidence on lipidomics and lipid-focused profiling in feline disease. By integrating findings across diverse clinical contexts, this review seeks to clarify current knowledge, identify consistent lipid alterations, and highlight priorities for future research in feline lipidomics.

## 2. Materials and Methods

This systematic review was conducted in accordance with the PRISMA 2020 (Preferred Reporting Items for Systematic Reviews and Meta-Analyses) guidelines [26]. 

### 2.1. Inclusion and Exclusion Criteria

Eligible studies were original research articles written in English, published in indexed journals between 1994 and 2026, and containing relevant information on lipid profile analysis in cats. Studies were excluded if they: (i) were not written in English; (ii) involved species other than cats; (iii) focused on microbiology or nutrition; (iv) were book chapters, technical manuals, conference proceedings, or abstracts; or (v) were duplicate records.

For mixed-species studies, only feline-specific data were extracted when clearly reported; studies in which cat data could not be disaggregated were excluded. Articles meeting the predefined criteria were evaluated in full, and screening and eligibility assessment were performed independently by two researchers.

### 2.2. Sources of Information and Search Strategy

A comprehensive literature search was conducted between 1 November 2024 and 1 March 2026 using the PubMed (National Library of Medicine, National Institutes of Health, Bethesda, MD, USA), ScienceDirect (Elsevier), and Scopus (Elsevier) databases. These databases were selected for their complementary coverage of biomedical, translational, and veterinary research. PubMed provides extensive indexing of peer-reviewed biomedical literature, ScienceDirect offers broad access to full-text articles in the life sciences, and Scopus ensures wider interdisciplinary coverage, including journals not indexed in PubMed. Together, these databases were considered sufficient to capture the majority of relevant studies on lipidomics and lipid-related profiling in feline disease.

The search strategy combined controlled vocabulary and free-text terms. The following search string was applied: (“cat” or “feline”) and (“lipidomics” or “lipid profile” or “lipidic profile” or “lipidome”). Searches were performed across all three databases, and filters were applied to include only articles written in English and published between 1994 and 2026.

Study selection, screening, and eligibility assessment were performed independently by two researchers. It is acknowledged that restricting the search to these three databases may have resulted in the omission of studies indexed exclusively in other sources. This is therefore considered a methodological limitation of the review and may have influenced the completeness of study retrieval, although it is unlikely to have significantly affected the overall conclusions.

### 2.3. Data Items

The primary data items extracted included lipid profile characteristics, fatty acid composition, lipid mediators (e.g., oxylipins), and lipoprotein distribution.

Additional variables included study design, sample size, biological matrices analysed, analytical methodologies, and clinical conditions evaluated.

No assumptions were made regarding missing data; only explicitly reported results were included.

A detailed summary of the included studies, methodologies, and key findings is provided in Table 1.

### 2.4. Risk of Bias in Individual Studies

The risk of bias for each included study was assessed using the Mixed Methods Appraisal Tool (MMAT), version 2018.

Case studies were evaluated using the screening questions for qualitative studies (Table 2). Descriptive cross-sectional studies were evaluated using the criteria for quantitative descriptive studies (Table 3). Case–control studies were evaluated using the criteria for quantitative non-randomized studies (Table 4).

All assessments were performed independently by two researchers, and disagreements were resolved by discussion. To avoid overly optimistic appraisal of small observational datasets, judgments were interpreted conservatively, particularly for studies with limited representativeness, small sample sizes, or incomplete reporting of potential confounders.

### 2.5. Data Analysis

Due to the heterogeneity of study designs, populations, and lipidomic methodologies, a quantitative meta-analysis was not performed. Instead, a structured narrative synthesis was conducted following principles aligned with the SWiM (Synthesis Without Meta-analysis) framework.

Studies were grouped according to disease category (e.g., hepatic disorders, urinary tract diseases, metabolic conditions), and key lipid alterations were summarized across studies. Emphasis was placed on identifying consistent patterns in lipid classes and metabolic pathways, including triglycerides, lipoproteins, phospholipids, sphingolipids, and oxylipins.

Where appropriate, differences in analytical platforms (e.g., targeted versus untargeted lipidomics and LC-MS-based approaches) and study design were considered when interpreting results. Given the variability across studies, findings were interpreted qualitatively, focusing on overall trends rather than direct comparisons of effect sizes.

Due to the limited number of included studies and the absence of quantitative synthesis, reporting bias was not formally assessed. Similarly, the certainty of evidence was not formally graded and was instead interpreted narratively based on study design, consistency, sample size, and methodological limitations.

No formal sensitivity analyses were conducted to assess the robustness of the synthesis, as no quantitative meta-analysis was performed and the included studies were limited in number and methodologically heterogeneous.

### 2.6. Registration and Protocol

This review was not registered. A protocol was not prepared before the study was initiated.

## 3. Results

A total of 349 records were identified across the three databases searched, with 221 records retrieved from ScienceDirect (63.3%), 67 records from PubMed (19.2%) and 61 records from Scopus (17.5%). After removal of 20 duplicate records, 329 articles were screened by title and abstract. Of these, 293 records were excluded for not meeting the predefined inclusion criteria, most commonly because the study species was not the cat, or because the articles focused on microbiology, nutrition, or were non-primary literature (chapters, manuals, conference proceedings, or abstracts).

**Figure 3:**
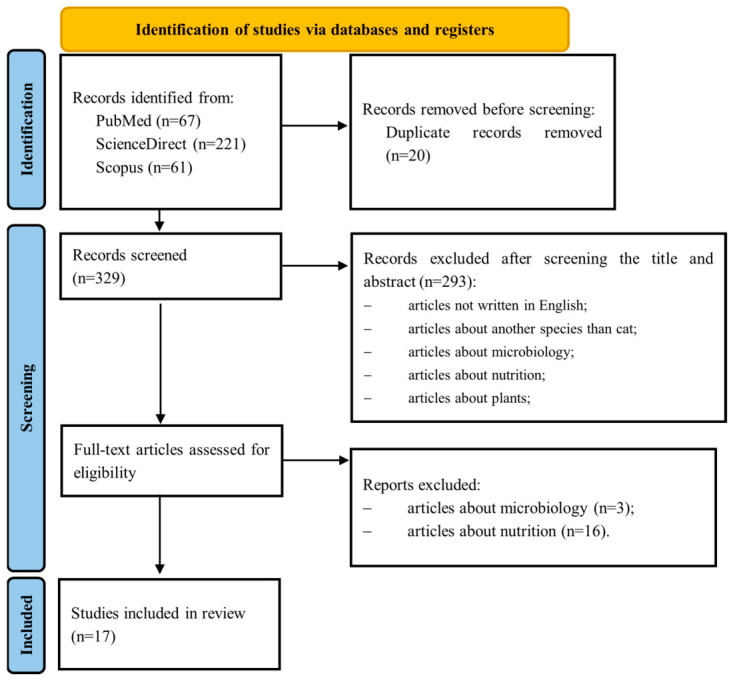
PRISMA 2020 based flow diagram of the identification, screening, and inclusion of studies in the systematic review.

A total of 36 full-text articles were assessed for eligibility. Following detailed evaluation, 19 articles were excluded (3 due to microbiology focus and 16 due to nutrition focus). Ultimately, 17 studies met all inclusion criteria and were incorporated into this systematic review (Figure 3).

The included studies varied considerably in design and scope and comprised case–control, cross-sectional descriptive, and case-based investigations. They also differed in clinical context, biological matrices analysed (blood, urine, effusions, liver tissue, kidney tissue, cerebrospinal fluid, tumour tissue, and cell lines), and analytical methodologies (ultracentrifugation, electrophoresis, gas chromatography, LC-MS/MS, DESI-MSI, LA-REIMS, and CLPDP). Given this heterogeneity, a descriptive synthesis was deemed the most appropriate approach (Table 1).

Across the 17 studies included in this review, lipidomics and lipid-focused profiling were applied to a broader range of clinical contexts, including hepatic, urinary, gastrointestinal, neurological, renal, oncological, and pharmacologically modulated states. Dietary conditions were inconsistently reported across the included studies, and in several cases were not controlled or explicitly described. This lack of dietary standardization may have influenced lipid profiles and represents an additional source of variability when comparing results across studies.

**Figure 4:**
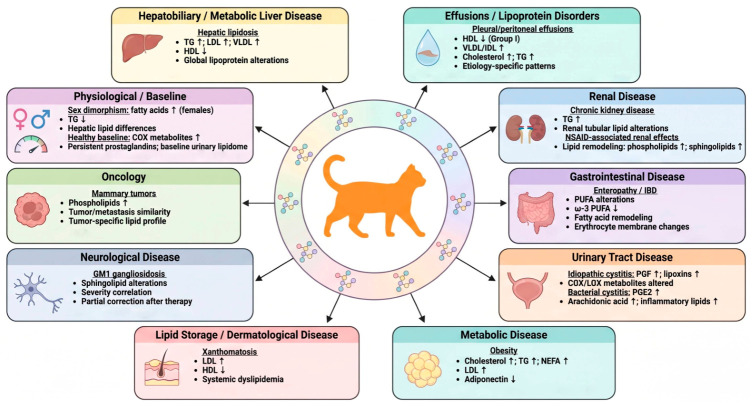
Overview of lipid and lipidomic alterations across feline diseases and physiological conditions. Schematic representation of major lipid changes reported in cats across multiple disease categories, including hepatobiliary, renal, gastrointestinal, urinary, metabolic, neurological, dermatological, and oncological conditions, as well as physiological modifiers. Key alterations in lipoproteins, fatty acids, phospholipids, sphingolipids, and bioactive lipid mediators are summarised, with arrows indicating direction of change (↑ increase; ↓ decrease). The central representation of the feline lipidome reflects the systemic and interconnected nature of lipid metabolism, providing an integrated overview of lipid dysregulation in feline pathophysiology [27,28,29,30,32,35,36,37,39,40,42,43,44,45,46,47].

Lipid alterations have been reported across multiple feline diseases and physiological conditions, involving distinct lipid classes and metabolic pathways (Figure 4).

The included studies were grouped into major clinical categories to facilitate interpretation of lipid-related findings across disease contexts. Key findings across disease categories are summarised below.

As illustrated in Figure 4, lipid alterations in cats extend across multiple disease categories, reflecting the systemic and interconnected nature of lipid metabolism. In pleural and peritoneal transudates, combined lipid and protein profiles revealed distinct patterns between underlying disease groups, although feline-specific interpretations remain constrained by the mixed-species design of the study [27].

Across hepatobiliary and metabolic conditions, particularly hepatic lipidosis, consistent disruptions in lipoprotein metabolism have been reported, indicating altered lipid transport and storage dynamics [28,29,30,31,32]. Similarly, in chronic enteropathies [33,34], disease-associated remodeling of fatty-acid composition highlights the involvement of membrane lipid turnover and inflammatory pathways [35].

Metabolic dysregulation was further evident in obesity, where lipid alterations are consistent with coordinated changes in energy balance, adipokine signaling, and lipoprotein kinetics [36,37,38]. In urinary tract disease, lipid mediator profiles were dominated by bioactive lipid mediators, highlighting the involvement of inflammatory pathways in both bacterial and idiopathic cystitis [39,40]. In parallel, sex-related differences in lipid profiles point to intrinsic physiological modulation of lipid metabolism, particularly at the hepatic level [32].

Recent studies have expanded the application of lipidomics to additional clinical contexts. In mammary oncology, largely overlapping lipidomic profiles between tumour and metastatic tissues suggest conserved metabolic reprogramming, with divergence emerging at the level of derived cell lines [41]. In renal tissue, the identification of species-specific lipid signatures suggests the presence of distinct metabolic adaptations within the feline kidney, with potential implications for chronic disease [42]. In GM1 gangliosidosis, lipid alterations closely tracked disease progression and therapeutic response, reinforcing the value of lipidomics in monitoring neurological disorders [43]. Pharmacological exposure also emerged as a relevant modifier, as repeated meloxicam administration induced systemic lipidomic changes across multiple biological compartments [44,45].

Finally, extreme dyslipidemic phenotypes, such as those observed in xanthomatosis and atherosclerosis, further illustrate the potential relevance of lipid imbalance in feline disease [46].

Collectively, these findings suggest that lipid remodeling may represent a recurring feature across feline pathophysiology, although evidence remains limited and heterogeneous.

**Table 1:** Simplified disease-oriented overview of the included evidence.

Study	Disease	Lipid Classes	Main Findings	Method	Potential Relevance	Key Caveat
Alonso 2022 [27]	Effusions (PLE, CKD, APSS, CVCS)	Lipoproteins, CHO, TG	HDL ↓ (Group I) VLDL/IDL ↑ Distinct disease patterns	Electrophoresis	Etiology differentiation	Mixed species sample
Blanchard 2004 [28]	Hepatic lipidosis	TG, LDL, HDL, VLDL	TG ↑ LDL ↑ VLDL ↑	Ultracentrifugation	HL diagnosis	Experimental model
Brociek 2026 [42]	Chronic Kidney Disease	TAG, MADAG	Unique renal tubular lipid species identified in cats	LC-MS	Species-specific renal lipid metabolism	Observational; causal relevance to CKD not established
Crisi 2024 [35]	Enteropathy	PUFA, FA	ω-3 PUFA ↓ FA remodeling	GC-FID	GI disease biomarker	No subtype discrimination
Gray-Edwards 2017 [43]	GM1 gangliosidosis	Sphingolipids	16 sphingolipid species altered Lipid levels correlated with disease severity Partial correction after gene therapy	LC-MS/MS	Neurological biomarker potential Therapeutic monitoring	Experimental disease model
Hoenig 2003 [36]	Obesity	CHO, TG, NEFA	CHO ↑ TG ↑ NEFA ↑	Ultracentrifugation	Metabolic insight	Small sample
Kobayashi 2020 [47]	Healthy baseline	Oxylipins, PUFA	COX metabolites ↑ PG persistent	LC-MS	Baseline lipidome	No diet control
Kobayashi 2021 [39]	Bacterial cystitis	PUFA, oxylipins	PGE2 ↑ AA ↑ Inflammatory markers ↑	LC-MS	Inflammation biomarker	Small sample
Minamoto 2019 [29]	Hepatic lipidosis	LDL, HDL	LDL ↑ HDL ↓	CLPDP	HL profiling	Heterogeneity
Mólnar 2024 [41]	Mammary tumors	Phospholipids	Tumor/metastasis similarity	DESI-MSI LA-REMIS	Tumour profiling	Mixed species
Muranaka 2011 [37]	Obesity	LDL, adiponectin	LDL ↑ Adiponectin ↓	Electrophoresis	Obesity grading	Cross-sectional
Pazak 1998 [30]	Hepatic lipidosis	TG, LDL, VLDL	TG ↑ LDL ↑ VLDL ↑	Ultracentrifugation	Pathophysiology insight	Old study
Rivera-Velez 2019a [44]	NSAID exposure (meloxicam)	Phospholipids	TG ↑ Phospholipid remodeling Drug-induced lipidomic changes	LC-MS	Drug-response lipidomic Biomarker discovery	Small sample size; short-term exposure
Rivera-Velez 2019b [45]	NSAID-associated renal effects	Phospholipids	Renal lipid remodeling TG ↑ Metabolic pathways altered	LC-MS	Mechanistic insight into NSAID nephrotoxicity	Small experimental sample
Takenouchi 2022 [40]	Idiopathic cystitis	COX/LOX metabolites	PGF ↑ Lipoxins ↑	LC-MS	Disease differentiation	Small sample
Valtolina 2017 [32]	Sexual dimorphism /HL	AA, TAG, SM	AA ↑ females TAG ↓	LC-MS/MS	Risk stratification	Small sample
Wisselink 1994 [46]	Xanthomatosis	LDL, HDL, VLDL	LDL ↑ HDL ↓	Electrophoresis	Atherosclerosis insight	Case study

Exploration of heterogeneity across the included studies indicated that between-study variation was mainly related to differences in study design, sample size, representativeness of the study populations, biological matrices analysed, analytical platforms, and degree of dietary and clinical standardization. More consistent patterns were observed in studies addressing hepatic lipidosis and lower urinary tract disease, whereas findings from mixed-species studies, experimental models, and small observational datasets were less directly comparable.

### Risk-of-Bias Analysis

The risk-of-bias analysis according to the Mixed Methods Appraisal Tool [48] is detailed in Table 2, Table 3 and Table 4.

**Table 2:** Evaluation of the quality of case study articles that were included using the Mixed Methods Appraisal [48].

Methodological Quality Criteria for Case Study Articles	Wisselink et al., 1994 [46]
Are there clear research questions?	Yes
Do the collected data allow to address the research questions?	Yes
Is the qualitative approach appropriate to answer the research question?	Yes
Are the qualitative data collection methods adequate to address the research question?	Yes
Are the findings adequately derived from the data?	Yes
Is the interpretation of results sufficiently substantiated by data?	Yes
Is there coherence between qualitative data sources, collection, analysis, and interpretation?	Yes

**Table 3:** Evaluation of the quality of cross-sectional articles that were included using the Mixed Methods Appraisal Tool [48].

Methodological Quality Criteria for Descriptive Cross-Sectional Study Articles	Alonso et al., 2022 [27]	Molnár et al., 2024 [41]	Brociek et al., 2026 [42]	Kobayashi et al., 2020 [47]
Are there clear research questions?	Yes	Yes	Yes	Yes
Do the collected data allow to address the research questions?	Yes	Yes	Yes	Yes
Is the sampling strategy relevant to address the research question?	Yes	Yes	Yes	Yes
Is the sample representative of the target population?	Yes	No	No	No
Are the measurements appropriate?	Yes	Yes	Yes	Yes
Is the risk of nonresponse bias low?	Yes	Yes	Yes	Yes
Is the statistical analysis appropriate to answer the research question?	Yes	Yes	Yes	Yes

**Table 4:** Evaluation of the quality of case–control articles that were included using the Mixed Methods Appraisal Tool [48].

Methodological Quality Criteria for Case–Control Articles	Blanchard et al., 2004 [28]	Minamoto et al., 2019 [29]	Pazak et al., 1998 [30]	Valtolina et al., 2017 [32]	(Crisi et al., 2024 [35]	Hoenig et al., 2003 [36]	Muranaka et al., 2011 [37]	Kobayashi et al., 2021 [39]	Takenouchi et al., 2022 [40]	Gray-Edwars et al., 2017 [43]	Rivera-Velez et al., 2019a [44]	Rivera-Velez et al., 2019b [45]
Are there clear research questions?	Yes	Yes	Yes	Yes	Yes	Yes	Yes	Yes	Yes	Yes	Yes	Yes
Do the collected data allow to address the research questions?	Yes	Yes	Yes	Yes	Yes	Yes	Yes	Yes	Yes	Yes	Yes	Yes
Are the participants representative of the target population?	Yes	Yes	Yes	Yes	Yes	Yes	Yes	No	No	No	No	No
Are measurements appropriate regarding both the outcome and exposure?	Yes	Yes	Yes	Yes	Yes	Yes	Yes	Yes	Yes	Yes	Yes	Yes
Are there complete outcome data?	Yes	Yes	Yes	Yes	Yes	Yes	Yes	Yes	Yes	Yes	Yes	Yes
Are the confounders accounted for in the design and analysis?	Yes	Yes	Yes	Yes	Yes	Yes	Yes	Yes	Yes	No	No	No
During the study period, did exposure occurred as intended?	Yes	Yes	Yes	Yes	Yes	Yes	Yes	Yes	Yes	Yes	Yes	Yes

Overall, the risk-of-bias assessment indicated moderate methodological limitations across the included studies (Table 2, Table 3 and Table 4). While most investigations clearly defined their objectives and used analytically appropriate methods, several studies were constrained by small sample sizes, experimental designs, limited representativeness, or highly specific disease models, which may reduce the generalizability of the findings.

In particular, studies rated as “No” for sample representativeness or participant selection may overestimate or underestimate lipid alterations, especially in heterogeneous clinical contexts or experimental settings. These concerns apply not only to observational feline disease studies, but also to pharmacological and translational models in which biological variability may not reflect routine clinical populations.

Taken together, the available evidence should be interpreted cautiously and considered exploratory, as the overall strength of evidence remains low to moderate due to small cohorts, observational or experimental designs, and methodological heterogeneity across studies. These limitations reinforce the need for larger, standardized, and better-controlled investigations to strengthen the robustness of lipidomic findings in feline disease research.

## 4. Discussion

Despite the breadth of reported lipid alterations, the available evidence remains limited by substantial heterogeneity in study design, analytical platforms, and population characteristics. Most studies were based on small sample sizes and employed diverse lipidomic or lipid profiling methodologies, which complicates direct comparison and limits the generalisability of findings. Furthermore, the lack of standardised protocols and reference intervals in feline lipidomics remains a major barrier to clinical translation.

**Figure 5:**
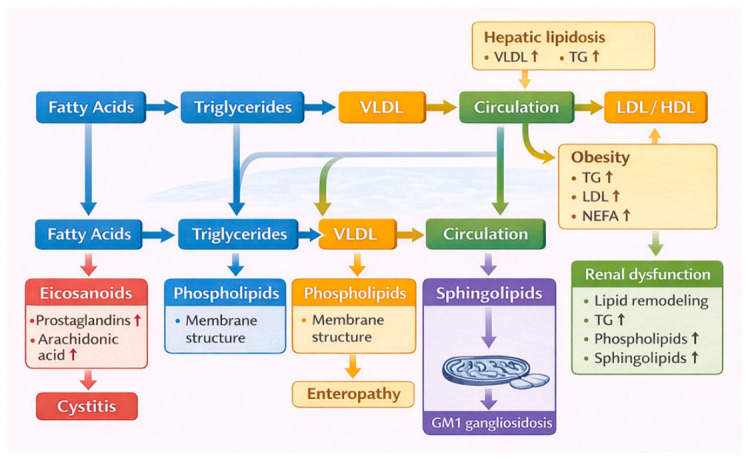
Simplified schematic of lipid metabolic pathways and disease-associated lipid alterations in cats. This diagram integrates findings from the reviewed studies to illustrate how alterations in major lipid classes, including fatty acids, triglycerides, lipoproteins, phospholipids, sphingolipids, and bioactive lipid mediators, are interconnected across metabolic pathways. Disease-associated changes are mapped onto key steps, highlighting the mechanistic links between lipid metabolism and feline pathophysiology. The arrows represent an increment in lipidic species.

The findings of this review suggest that lipid alterations in cats are not isolated events but may provide complementary insights alongside conventional metabolic assessments in research settings. This integrative perspective is summarised in Figure 5.

Reference lipid profiles in healthy cats remain insufficiently characterized. Kobayashi [47] provided an initial description of urinary lipid mediators; however, the study included only five neutered cats and did not report strict dietary control. Given that diet, body condition, sex, and reproductive status can influence lipid composition, these baseline data should be interpreted as preliminary. Valtolina [32] further demonstrated that sex-related differences, particularly in arachidonic acid-containing lipids, may be biologically relevant, reinforcing the need for stratified reference datasets in future studies.

Across the seventeen included studies, lipid-focused approaches identified condition-specific alterations in lipid classes, lipoprotein distribution, fatty acid composition, sphingolipid metabolism, and oxylipin biosynthesis. These findings further support the relevance of lipid metabolism in feline pathophysiology, although their interpretation must remain cautious due to the exploratory nature of the available evidence.

The mixed-species effusion study by Alonso [27] suggests that lipoprotein patterns in body fluids may assist in differentiating pleural and peritoneal transudates (although this application remains preliminary), particularly in diagnostically ambiguous cases. However, the inclusion of both canine and feline samples and the heterogeneity of disease groups limit direct clinical applicability in cats. At present, conventional parameters such as total protein remain more robust diagnostic indicators, with lipid profiling representing a potential complementary tool rather than a primary diagnostic approach [49,50,51].

Sexual dimorphism represents an additional source of physiological variability. Valtolina [32] reported significant differences in hepatic and plasma lipid profiles between male and female cats, including higher arachidonic acid levels in intact females. These findings align with observations in other species and underscore the importance of incorporating sex-specific considerations into feline lipidomic research and interpretation.

Hepatic lipidosis is the condition in which lipid alterations are most consistently described in cats. Across multiple studies [28,29,30,31,32], characteristic features include triacylglycerol accumulation, altered VLDL handling, changes in LDL and HDL composition, and modifications in sphingolipid profiles. These findings collectively suggest a disruption of hepatic lipid trafficking processes rather than a single isolated metabolic defect. Nevertheless, mechanistic interpretation should remain cautious. While several pathways—such as impaired lipoprotein export, altered fatty acid flux, and regulatory limitations in VLDL metabolism—are supported by feline data, broader interpretations often rely on extrapolation from other species and general lipid biology. Accordingly, hepatic lipidosis represents the most robust example of disease-associated lipid remodeling in cats, but not yet a fully resolved mechanistic framework.

Obesity is a prevalent metabolic disorder in cats and is associated with systemic inflammation, insulin resistance, and altered lipid metabolism. Muranaka [37] demonstrated that cholesterol-rich lipoprotein profiles are consistent with early metabolic alterations, including increased LDL and reduced adiponectin concentrations. Similarly, Hoenig [36] reported increased VLDL turnover and altered insulin secretion patterns in obese cats, resembling features of insulin dysregulation described in other species. Although these findings are broadly consistent with metabolic dysfunction, they do not yet support the use of lipid-based parameters as validated clinical biomarkers. Instead, they suggest that lipid-focused measurements may complement conventional metabolic assessments in research settings. Larger, longitudinal, and diet-controlled studies are required to determine whether these lipid alterations have diagnostic or prognostic utility [52].

The evidence for lipid profile alterations in feline chronic enteropathy is currently limited to a single study of erythrocyte membrane fatty acids [35]. This study reported alterations in omega-3 PUFA composition, suggesting systemic lipid remodeling associated with intestinal disease. However, the lack of clear subtype differentiation, combined with potential dietary and case-selection confounders, limits the generalizability of these findings. Rather than establishing a diagnostic role, these results indicate that chronic enteropathies may exert measurable systemic effects on lipid metabolism, thereby supporting the rationale for future studies integrating lipidomic approaches with detailed clinical phenotyping and dietary control.

Urinary lipid mediators represent a promising area of investigation in the context of lower urinary tract disease, as they provide a non-invasive window into inflammatory and epithelial processes. The reviewed studies indicate that distinct oxylipin and prostaglandin profiles are associated with bacterial and idiopathic cystitis [39,40,53]. However, the small sample sizes, variability in urine collection methods, and limited control of confounding factors such as diet, medication, and urinary pH constrain interpretation. These findings should therefore be regarded as preliminary biomarker signals rather than clinically applicable diagnostic tools.

Recent studies further extend the application of lipidomics beyond classical metabolic and inflammatory conditions. In oncology, lipidomic signatures appear to be preserved across tumour progression and metastasis, suggesting potential translational relevance in feline mammary neoplasia, although feline-specific interpretation remains limited by mixed-species design and ex vivo modelling [41].

Renal lipid metabolism in cats may also present distinctive biological features. Brociek [42] identified unusual lipid species in feline renal tubular cells, including monoalkyl-diacylglycerols, thereby challenging the traditional interpretation of renal lipid accumulation as merely incidental and suggesting relevance to feline chronic kidney disease [23].

In neurological disease, targeted lipidomic analysis in GM1 gangliosidosis demonstrated that sphingolipid alterations correlate strongly with disease progression and therapeutic response, supporting the potential of lipidomic approaches as a research tool for monitoring disease progression in feline neurodegenerative disorders [43].

Pharmacological modulation of the lipidome should also be considered when interpreting feline lipidomic data. Repeated meloxicam administration significantly altered lipid profiles in plasma, urine, and renal tissue, indicating that drug exposure can act as a major biological modifier and potential confounder in lipidomic studies [44,45].

Finally, the reported case of xanthomatosis and atherosclerosis [46] illustrates the potential severity of dyslipidemia in cats and highlights the potential role of lipid profiling in identifying extreme metabolic disturbances [54]. However, case-level evidence does not permit conclusions regarding prevalence, causality, or biomarker performance. Instead, it emphasizes the need for further investigation into genetic, metabolic, and environmental contributors to dyslipidemia in feline populations.

### 4.1. Integrative Interpretation

This systematic review indicates that characterization of lipid profiles in feline cells, tissues, and biological fluids provides meaningful insights into physiological variation and disease-associated metabolic remodeling across a range of clinical contexts, including metabolic, inflammatory, renal, neurologic, oncologic, and pharmacologically modulated states.

Across the included studies, recurring patterns of alteration were observed, particularly involving lipoprotein redistribution, changes in fatty-acid composition, sphingolipid remodeling, and oxylipin biosynthesis. These findings collectively support the biological relevance of lipid metabolism in feline pathophysiology, especially in hepatic lipidosis, obesity-associated metabolic disturbance, lower urinary tract disease, renal lipid handling, and neurological lipid storage disorders.

However, the available evidence remains heterogeneous, methodologically constrained, and largely exploratory. Lipidomics and lipid-focused profiling should therefore not yet be interpreted as established diagnostic or prognostic tools in feline clinical practice. Rather, they represent a promising research framework with potential to support biomarker discovery, mechanistic clarification, and improved disease stratification as the field matures.

### 4.2. Future Directions

Advancing this field will require larger and more representative cohorts, clearer and standardized phenotyping, and study designs that account for breed- and sex-related metabolic variability. Improved standardization of pre-analytical procedures, including sample collection, handling, and storage, will be essential to reduce variability across studies.

Harmonization of analytical methodologies is also critical. High-resolution LC-MS/MS-based lipidomic approaches—combining discovery-oriented untargeted workflows with targeted validation panels—appear particularly promising for future feline research. These strategies may enable both comprehensive lipidome mapping and the development of reproducible biomarker panels.

Furthermore, integration of lipidomics with complementary multi-omics approaches, such as transcriptomics, proteomics, and metabolomics, together with detailed dietary and environmental metadata, is likely to enhance mechanistic interpretation and translational relevance. Such integrative strategies may ultimately facilitate the transition from exploratory lipid profiling to clinically actionable applications.

### 4.3. Limitations

Several limitations should be considered when interpreting this systematic review. First, the number of available studies on feline lipidomics and lipid-focused profiling remains limited, thereby restricting the breadth of comparison and precluding quantitative synthesis. Second, the search strategy was confined to three major databases (PubMed, ScienceDirect, and Scopus). Although these sources were considered sufficient to capture the core identifiable literature, relevant studies indexed elsewhere may not have been retrieved. Importantly, no additional eligible feline studies were identified outside these databases during the revision process.

Third, the included evidence is methodologically heterogeneous, with substantial variation in analytical platforms, lipid classes quantified, biological matrices examined, and reporting formats. In addition, most studies involved small sample sizes, and some lacked representative sampling, limiting both statistical power and generalizability.

Specific methodological limitations were also identified within individual studies. For example, in the baseline urinary study, dietary intake was not strictly standardized, which is particularly relevant given the known influence of nutrition on lipid mediator profiles and its potential impact on generalizability.

At the review level, the absence of protocol registration and the lack of a predefined protocol reduce methodological transparency. Moreover, reporting bias and certainty of evidence were not formally assessed using dedicated frameworks. While these limitations do not negate the value of the review, they necessitate cautious interpretation of its conclusions.

## 5. Conclusions

Despite these limitations, the present review consolidates a previously fragmented body of literature and highlights the most consistent and biologically relevant lipid alterations identified to date. It also identifies key priorities for future research, including improved standardization, stronger validation, and clearer distinction between exploratory lipidomics and clinically relevant biomarker research.

Overall, lipid alterations provide biologically informative insights into feline disease processes. However, the current evidence remains heterogeneous and largely exploratory. The clinical applicability of these findings for diagnostic or prognostic use remains to be established and requires further validation in larger, standardised studies.

## Data Availability

All information collected within the scope of this work is included in the review.

## Acronyms and Abbreviations

AA	arachidonic acid
ALB	albumin
APSS	acquired portosystemic shunt
CHO	cholesterol
CKD	chronic kidney disease
CLPDP	continuous lipoprotein density profile
COX	cyclooxygenase
CVCS	caudal vena cava syndrome
CYP	cytochrome P450
DESI-MSI	Desorption Electrospray Ionization–Mass Spectrometry Imaging
DHA	docosahexaenoic acid
DHET	dihydroxyeicosatrientic acid
EPA	icosapentaenoic acid
FA	fatty acid
FFA	free fatty acid
FHL	feline hepatic lipidosis
FIC	feline idiopathic cystitis
HDL	high-density lipoprotein
IDL	intermediate-density lipoprotein
IFHL	idiopathic feline hepatic lipidosis
LA	linoleic acid
LA-REIMS	Laser-Assisted Rapid Evaporative Ionisation Mass
LDL	low-density lipoprotein
LOX	lipoxygenase
MADAG	Monoalkyl-diacylglycerol
NEFA	non-esterified fatty acid
PGB	prostaglandin B
PGD	prostaglandin D
PGE	prostaglandin E
PGF	prostaglandin F
PL	phospholipid
PLE	protein-losing enteropathy
PUFA	polyunsaturated fatty acid
RBC	red blood cell
SM	sphingomyelin
TAG	triacylglycerol
TP	total protein
TRL	triglyceride-rich lipoprotein
VLDL	Very low-density lipoprotein
